# Synergistic gastroprotective activity of methanolic extract of a mixture of *Melastoma malabathricum* and *Muntingia calabura* leaves in rats

**DOI:** 10.1186/s12906-017-1992-9

**Published:** 2017-11-09

**Authors:** Siti Zawanah Halim, Zainul Amiruddin Zakaria, Maizatul Hasyima Omar, Norhafizah Mohtarrudin, Ikarastika Rahayu Abdul Wahab, Muhammad Nazrul Hakim Abdullah

**Affiliations:** 1 Department of Biomedical Science, Faculty of Medicine and Health Sciences, Universiti Putra Malaysia, 43400 Serdang, Selangor Malaysia; 2 Integrative Pharmacogenomics Institute (iPROMISE), Level 7, FF3 Building, Universiti Teknologi MARA, 42300 Puncak Alam, Selangor Malaysia; 3 Halal Product Research Institute, Universiti Putra Malaysia, 43400 Serdang, Selangor Malaysia; 4 Phytochemistry Unit, Herbal Medicine Research Centre, Institute for Medical Research, 50588 Jalan Pahang, Kuala Lumpur Malaysia; 5 Department of Pathology, Faculty of Medicine and Health Sciences, Universiti Putra Malaysia, 43400 Serdang, Selangor Malaysia; 6 Faculty of Agro-based Industry, Universiti Malaysia Kelantan, Jeli Campus, 17600 Jeli, Kelantan Malaysia

**Keywords:** *Melastoma malabathricum*, *Muntingia calabura*, Synergistic effect, Gastric ulcer, Antisecretory, Antioxidant

## Abstract

**Background:**

*Melastoma malabathricum* L. (family Melastomaceae; MM) and *Muntingia calabura* L. (family Elaeocarpaceae; MC) have been separately reported to possess gastroprotective activity. In an attempt to develop a pharmaceutical product with antiulcer potential, the synergistic gastroprotective activity of methanolic extract of a mixture of MM and MC (MMMC) at various ratios was evaluated in rat models.

**Methods:**

Rats were pre-treated orally with 2% Tween 80 (vehicle), 100 mg/kg ranitidine (reference drug) or MMMC (ratios of 1:1, 1:3 and 3:1 (*v*/v); doses of 15, 150 or 300 mg/kg) and then subjected to the ethanol-induced gastric ulcer or pyloric ligation assays. Stomach of rats from the former assay was collected and subjected to the macroscopic and microscopic observations, and enzymatic and non-enzymatic antioxidant studies while the gastric juice content and tissue from the latter assay were subjected to the antisecretory activity study. The UHPLC analysis of MMMC was also performed.

**Result:**

MMMC, in the ratio 1:1, demonstrated the most effective (*P* < 0.001) gastroprotective activity indicated by the highest reduction in ethanol-induced ulcer area formation. These macroscopic findings were supported by the microscopic observations. Except for pH and total acidity, MMMC also significantly (*P* < 0.001) reduced the volume of gastric content but increased the gastric wall mucus content in the pyloric-ligation test. MMMC also demonstrated remarkable antioxidant activity indicated by the highest total phenolic content (TPC) value and oxygen radical absorbance capacity (ORAC) activity with the recorded IC_50_ value of approximately 53 μg/mL for the 2,2- diphenyl-1-picrylhydrazyl (DPPH) scavenging activity. MMMC also improved the catalase (CAT), superoxide dismutase (SOD), glutathione (GSH), prostaglandin E2 (PGE2) and malondialdehyde (MDA) activities of the gastric tissue intoxicated by ethanol. UHPLC analysis of MMMC confirmed the presence several flavonoid-based bioactive compounds.

**Conclusion:**

MMMC, at the ratio of 1:1 (*v*/v), exerts gastroprotective activity partly by activating its antisecretory and antioxidant activities, and via modulation of the gastric tissue endogenous antioxidant system.

## Background

One of well-known disease that had affected nearly 8-10% of the global population is peptic ulcer [1], and of these numbers, 5% suffer from gastric ulcers [2]. Diverse factors such as over-consumption of alcohol, a tough lifestyle, usage of steroidal and non-steroidal anti-inflammatory drugs (NSAIDs) and drugs which stimulate gastric acid and pepsin secretion, *Helicobacter pylori* infections and smoking contribute to the pathogenesis of gastric ulcers [3]. An imbalance between the aggressive factors such as acid and pepsin secretion, *H. pylori,* refluxed bile, deliverance of leukotrienes and reactive oxygen species (ROS) and mucosal protective factors that include bicarbonate secretion, mucus-bicarbonate barrier, surface active phospholipids, prostaglandins (PGs), mucosal blood circulation, cell renewal and relocation, non-enzymatic and enzymatic antioxidants and some growth factors [4] leads to gastric damages.

The prevention or cure of peptic ulcers has become a foremost challenge in the current medicine world. Although treatment of peptic ulcer depends on the cause and may need antibiotics to kill *H. pylori*, secretion of gastric acid is still believed to remain as the central component of this disease. Thus, inhibition of gastric acid secretion is the key therapeutic target for ulcer diseases [5]. Therefore, current medicinal treatment of gastric ulcers that convenient is generally based on the inhibition of gastric acid secretion by histamine H_2_-antagonists, proton pump inhibitors, and antimuscarinics, as well as on acid-independent therapy provided by sucralfate and bismuth cholinergic [6]. However, gastric ulcer therapy faces a major drawback nowadays as most of the drugs available in the market are often associated with side effects [3, 7].

The used of animal assays, particularly rats, to study the gastroprotective potential of medicinal plants are necessary as the physiology of the rat’s stomach, which is also classified as a mammal, closely resembles that of the human. The exposure of rats to certain chemicals/drugs, in this case, ethanol has been known to induce gastric mucosa damage leading to gastric ulcer formation and by oral administering the plant’s extract to the rats, the application of medicinal plants via traditional oral consumption can be achieved. Moreover, part of the mechanisms of antiulcer activity exerted by the plant’s extract can be determined via the pylorus ligation assay in rats. Through this assay the ability of the extract to affect several parameters of the gastric juice content (i.e. pH, volume and acidity) and the volume of gastric wall mucus content can be determined. In addition, the role of the endogenous antioxidant system in protecting the stomach from the toxic effect of chemicals and the ability of plant’s extract to trigger this system can also be measured directly from the collected stomach.

In this context, the use of medicinal plants has gained interest of many researchers. The natural products field is in continuous expansion all over the world and became an attractive source of new drug for the treatment and prevention of many diseases. A diverse range of bioactive molecules isolated from plant natural products has been shown to produce promising results for the treatment of gastric ulcer [8].

Two of plants that are currently under investigation for their potential pharmacological activities in our laboratory are *Melastoma malabathricum* L. (family Melastomataceae) and *Muntingia calabura* L. (family Muntingiaceae). Also known as *senduduk ungu* in Malaysia, *M. malabathricum* is one of the most common herbs or small shrubs found throughout the tropic area especially in the moist land mostly from Indian Ocean Islands, Taiwan and Australia [9]. The leaves, shoots, barks, seeds and roots of *M.malabathricum* have been used as a folk remedy to treat diarrhea, dysentery, hemorrhoids, cuts and wounds, toothache, and stomachache [9]. Scientific evaluations on *M. malabathricum* have revealed several pharmacological activities possessed by the plant. *M. malabathricum* leaves have been reported to exhibit significant antinociceptive, anti-inflammatory, wound healing, cytotoxic, antidiarrheal and antioxidant activities [9]. Flavonoids, tannins, saponins, triterpenes and steroids have been detected in the leaves of *M. malabathricum* [10].

On the other hand, *M. calabura*, commonly known as Jamaican cherry or locally known as *kerukup siam* in Malaysia, is widely cultivated in the warm areas of Asian region, including Malaysia [11]. In Asia and tropical America, different parts of this tree have been documented for several medicinal uses. The leaves, flowers, barks and roots of *M. calabura* have been used as a folk remedy to treat migraine, fever and incipient cold. Besides, they are also employed as antiseptic, antispasmodic, and antidyspeptic agent [12, 13]. It also has been scientifically validated to possess several pharmacological activities. Significant antinociception [14–16], antitumor [12, 17], anti-inflammatory, antipyretic [16], antibacterial [18], antiproliferative and antioxidant [19] activities have been exhibited by the leaves of *M. calabura.* Several types of flavonoids have been isolated and identified from the leaves, roots and stem barks of *M. calabura* [12, 13, 17, 20, 21].

Our previous studies have reported on the significant antiulcer activity of methanol extract of the respective *M. malabathricum* and *M. calabura* leaves using several animal models and its association with the antioxidant and anti-inflammatory activities of each plant [22, 23]. Therefore, in an attempt to develop a pharmaceutical product with antiulcer potential from these two plants, the present study aimed to determine their synergistic effect at various ratio following subjcection to several gastro-intoxicated assays.

## Methods

### Plant material

Both *M. malabathricum* and *M. calabura* were collected from different localities around Selangor, Malaysia. The plants were identified by a botanist and voucher specimens for *M. malabathricum* (SK2684/15) and *M. calabura* (SK2683/15) have been deposited at the Institute of Bioscience, UPM, Selangor, Malaysia.

### Preparation of methanol extract of *M. malabathricum* and *M. calabura* (MMMC)

The preparation of MMMC was done according to Zakaria et al. [19]. Matured leaves were ground into powder after air-drying them at room temperature (27 ± 2 °C) for 1-2 weeks. Then, the mixture of leaves powder of *M. malabathricum* and *M. calabura*, in the ratio of 1:1, 1:3 or 3:1 (*v*/v), were immersed in methanol in the ratio of 1:20 (*w*/*v*) for 72 h. Each supernatnant obtained was filtered using cotton wools followed by Whatman no.1 filter papers. The soaking and filtration processes were repeated on the residue for another two times. The collected filtrate from each soaking process was pooled together and evaporated using the rotary evaporator at 40 °C under reduced pressure.

### In vitro antioxidant activity of MMMC

#### DPPH radical scavenging activity

Antioxidant reducing activity on DPPH radical was carried out according to the method of Lai et al. [24] with a slight modification. The sample of MMMC (200 μL) was mixed with 800 μL of 100 mM Tris-HCl buffer, pH 7.4. The mixture was then added to 1.0 mL of 500 μM DPPH (previously prepared in methanol). This was made up to the DPPH final concentration of 250 μM. The control was conducted by mixing 200 μL of methanol with 1.0 mL DPPH. The mixture was then shaken vigorously and left to stand for 20 min at room temperature in a dark room. The absorbance was read using a UV-vis spectrophotometer at 517 nm with methanol as the blank. Triplicate measurements were carried out and their activity was calculated based on the percentage of scavenged DPPH as follows:

Scavenging activity (%) = [1 - (Absorbance of sample at 517 nm / Absorbance of control at 517 nm)] × 100.

### Total phenolic content (TPC)

The content of reducing components (expressed as TPC) was estimated using the Folin-Ciocalteau assay according to a method developed by Velioglu et al. [25] with a slight modification. Briefly, 0.75 mL of 10-fold diluted Folin-Ciocalteu reagent and 100 μL of methanolic extract were placed in a test tube. The mixture was mixed and allowed to stand at room temperature for 5 min. Then, 0.75 mL of 6% (*w*/*v*) sodium carbonate solution was added. The mixture was homogenized and let to stand at room temperature for 90 min. TPC was determined using a Spectronic Genesis™ spectrophotometer at 725 nm. The standard calibration curve was schemed using gallic acid at the concentrations of 0.02-0.1 mg/mL. The TPC was expressed as gallic acid equivalent (GAE) mg/100 g edible portion.

### Oxygen radical absorbance capacity (ORAC)

The ORAC assay was performed as described by Huang et al. [26] with some modifications. A fresh 2,2′-azobis 92-methylpropionamidine dihydrochloride (AAPH) (0.65 g) was dissolved in 10 mL of 75 mM phosphate buffer (pH 7.4) to a final concentration of 240 mM. A fresh fluorescein stock solution (1 mM) was made in 75 mM phosphate buffer (pH 7.4) to a final concentration of 240 mm. A fluorescein stock solution (1 mM) was made in 75 mM phosphate buffer (pH 7.4) and stored, wrapped in foil at 5 °C. Immediately prior to use, the stock solution was diluted 1:100,000 with 75 mM phosphate buffer. The diluted sodium fluorescein was made fresh daily. The sodium fluorescein solution (150 μL) was added to the interior experimental wells. The blanks received 25 μL of Trolox dilution. The sample wells received 25 μL samples. The plate was then allowed to equilibrate by incubating for 10 min at 37 °C. The BMG Omega Fluostar Fluorescent Spectrophotometer with injector was used with an excitation filter of 485 nm bandpass and emission filter of 528 nm bandpass. The plate reader was controlled by MARS data analysis software. Reaction was initiated by the addition of 25 μL of AAPH solution (240 mM) using the microplate reader’s injector for a final reaction volume of 200 μL. The addition of 25 μL of AAPH solution was followed by shaking at maximum intensity for 50 s. The fluorescence was then monitored kinetically with data taken every minute. The fluorescence of each well was measured by top reading every 60 s. ORAC values were calculated using MARS Data Analysis Reduction Software.

### Animals

All experiments were performed on male Sprague-Dawley rats (180–200 g; 8-10 weeks old) obtained from the Animal Unit, Faculty of Medicine and Health Sciences, UPM, Malaysia. The animals were caged in polypropylene cages with wood shaving, fed with standard pellet and enabled free access to water. They were kept at room temperature (27 ± 2 °C; 70-80 humidity 12 h light/darkness cycle) in the Animal Holding Unit (UPM). The rats were fasted for 48 h prior to all assays. The standard drugs and MMMC were administered orally (p.o) by gavage for seven consecutive days with 2% Tween 80 (10 ml/kg) as the vehicle. The use of animals in the following study was supported by the Animal Care and Used Committee (ACUC) of the Faculty of Medicine and Health Sciences, UPM (approval no. UPM/IACUC/AUP-R010/2015).

### Determination of antiulcer activity of MMMC at various ratios using the ethanol-induced gastric ulcer assay

The experiment was performed according to the methods described by Balan et al. [22]. The rats were divided randomly into 11 groups (*n* = 6) and orally administered once daily for 7 consecutive days with the respective test solution namely vehicle (2% tween 80, 10 mL/kg), ranitidine (100 mg/kg) or different ratio (1:1, 1:3 and 3:1 (*v*/v)) of MMMC, each prepared in the doses 15, 150 and 300 mg/kg). On Day 7th, after a total of 48 h fasting, the rats were given absolute ethanol (5 mL/kg) after 1 h of treatment in order to induce gastric ulceration. The animals were anesthetized by diethyl ether and euthanized by cervical dislocation 1 h after the ulcer induction. The stomachs were removed and opened along the greater curvature to determine the lesion damage. Every opened and spread-out stomach was photographed and the ulcer area was quantified by superimposing transparent grid paper with minimum square equal to 1 mm^2^ [27]. The ulcer area (UA) in mm^2^ was determined for each stomach in the group. Percentage protection provided by the fractions was calculated using the following formula:

$$ \mathrm{Protection}\kern0.5em \left(\%\right)=\frac{\mathrm{UA}\kern0.5em \mathrm{control}-\mathrm{UA}\kern0.5em \mathrm{pre}-\mathrm{treated}\kern0.5em \mathrm{group}}{\mathrm{UA}\;\mathrm{control}}\times \kern0.5em 100 $$

### Histopathological studies of ulcer-induced gastric tissues pretreated with MMMC at various ratios

The samples of gastric tissue from each group were collected and fixed in 10% formalin. The samples were then embedded in paraffin after the tissue was processed. This was followed by sectioning (3-5 μm) and staining with hematoxylin and eosin dye. The sections were viewed and analyzed using light microscopy and photographed.

### Determination of effects of MMMC at various ratios on several parameters indicator of gastric ulcer formation using the pylorus ligation assay

Pylorus ligation was carried out according to the method described by Shay [28] with slight modifications. Sixty-six rats were divided into 11 groups. Group-I (control) was treated with vehicle (2% Tween 80), Group-II (positive control) was given 100 mg/kg ranitidine (p.o) while Group-III until Group- XI were treated with different ratios of MMMC (1:1, 1:3 and 3:1; *v*/v) prepared in the dose of 15, 150 and 300 mg/kg, respectively. The pylorus ligation procedure was performed 1 h after the last administration of the respective test solutions on 48 h fasted rats. Under light anesthesia induced by ketamine HCl (100 mg/kg, intramuscular) and xylazine HCl (16 mg/kg, intramuscular), a 2 cm long incision was made in the abdomen just below the sternum. The stomach was exposed, and a thread was placed around the pyloric sphincter and tied in a tight knot. Care was taken while tying the knot to avoid involving blood vessels in the knot. The abdomen was sutured, and the skin was cleared from any blood spots or bleeding. The animals were sacrificed 6 h after the procedure of pylorus ligation by cervical dislocation. The stomachs were removed, and the contents were drained out, collected, and centrifuged. The stomach was opened along the greater curvature to discover the lesion damage as described by Balan et al. [22]. The percentage protection was calculated using the following formula:

$$ \mathrm{Protection}\kern0.5em \left(\%\right)=\frac{\mathrm{UA}\kern0.5em \mathrm{control}-\mathrm{UA}\kern0.5em \mathrm{pre}-\mathrm{treated}\kern0.5em \mathrm{group}}{\mathrm{UA}\;\mathrm{control}}\times \kern0.5em 100 $$

### Determination of volume, pH and, free and total acidity of gastric content

The extracted gastric content was centrifuged at 2500 rpm for 10 min. The volume and pH of the gastric juice were measured and were subjected to free and total acidity estimation. The method described by Srivastava et al. [29] was employed in free and total acidity estimation. Free acidity was determined by titration with 0.01 N NaOH with methyl orange reagent until the color of the solution became yellowish. The volume of alkali added was recorded. Then, two to three drops of phenolphthalein were added and the solution was titrated until a definite red tinge appears. The total volume of NaOH added was noted and this matches to total acidity. Acidity was calculated using the following formula:

$$ \mathrm{Acidity}\;\left(\mathrm{meq}/1\right)=\frac{\mathrm{Volume}\  \mathrm{of}\kern0.5em \mathrm{NaOH}\kern0.5em \times \kern0.5em \mathrm{normally}\kern0.5em \mathrm{of}\kern0.5em \mathrm{NaOH}\kern0.5em \times \kern0.5em 100}{0.1} $$

### Estimation of gastric wall mucus content

Gastric wall mucus content was determined by the method described by Corne et al. [30] with slight modifications. The stomach was opened along the greater curvature, weighed, and immersed in 10 ml of 0.1% Alcian Blue in 0.16 M sucrose/0.05 M sodium acetate, pH 5.8 for 2 h. The excessive dye was then removed by two successive rinses in 0.25 M sucrose solution (15 min each). The remaining dye complexed with the gastric mucus were extracted with 0.5 M MgCl_2_ for 2 h and shaken intermittently for 1 min in every 30 min interval. The blue extract was then shaken vigorously with an equal volume of diethyl ether and the outcoming emulsion was centrifuged at 3600 rpm for 10 min. The OD of Alcian Blue in the aqueous layer was read at 580 nm using a spectrophotometer. The quantity of Alcian Blue extract per gram wet stomach was then determined from a standard curve.

### Effect of MMMC on the superoxide dismutase (SOD), catalase (CAT) and glutathione (GSH) activities in the ethanol-induced gastric ulcer tissue

To determine the SOD, CAT and GSH activities in ethanol-induced gastric ulcer tissue following pretreatment with MMMC, the assays performed by Leyck and Parnharm [31] was followed with slight modification. The gastric ulcer tissue was homogenized in 1.15% potassium chloride at the ratio of 1:5 (*w*/*v*) followed by centrifugation for 15 min at 4 °C. The supernatant was collected and the level of SOD, CAT and GSH activities was measured using the respective Superoxide Dismutase Assay, Glutathione Assay and Catalase Assay Kit (Cayman Chemical Company, USA) according to the manufacturer’s instructions. The optical densities of SOD, GSH and CAT were measured using the ELISA Reader (Asys UVM 340, UK) at 440, 405 and 540 nm, respectively.

### Measurement of malondialdehyde (MDA) level

The ethanol-induced gastric ulcer tissue samples were homogenized in 1.15% potassium chloride at the ratio of 1:5 (*w*/*v*) followed by centrifugation at 4 °C. The supernatant of each sample was collected and subjected to the MDA activity measurment using a kit from Cayman Chemical Company (USA). The optical density was measured between 530 to 540 nm using an ELISA reader (Asys UVM 340, UK). The results were expressed as ng/mL protein.

### Prostaglandins E_2_ (PGE_2_) determination

A PGE_2_ determination is an assay which detects the reaction between PGE_2_ and PGE_2_-acetylcholinesterase (AchE) conjugate, identified as the PGE_2_ tracer. The supernatant obtained following the homogenization and centrifugation of gastric ulcer tissue samples were subjected to PGE_2_ activity determination using the PGE_2_ kit from Cayman Chemical Company (USA). Using the ELISA reader (Asys UVM 340, UK), the absorbance that represents the PGE_2_ activity was determined at the wavelength of 405 and 420 nm. The results were expressed as pg/mL protein.

### UHPLC-ESI-MS analysis of MMMC at the ratio of 1:1 (*v*/v)

The UHPLC-ESI-MS system consisted of Dionex Ultimate 3000 series including a binary pump with a built in solvent degasser, a diode-array detector, an autosampler equipped with a column oven and a column compartment (Thermo Fisher Scientific, San Jose, CA, USA). The MMMC was separated on a Cortecs C18 column (1.6 μl, 2.1 × 50 mm I.D.; Waters Co.. Milford, MA, USA) maintained at 40 °C. The mobile phase consisted of a mixture 0.1% formic acid in water and a mixture 0.1% formic acid in acetonitrile. A constant flow of 0.3 ml/min was applied. The acetonitrile percentages were: 0-5 min, 20%; 5–17 min, linearly from 20% to 60%; 17-20 min, 90%; 20 – 22 min, linearly from 90% to 5%; 22-30 min, (re-equilibration step), 5%. The effluent from the chromatographic column was injected (10 μl) into a linear Q Exactive ion-trap-Orbitrap mass spectrometer (Thermo Fisher Scientific, USA) equipped with an electrospray ionization (ESI) interface in the negative ion mode. The mass recognization was performed in a range of 150-1500 m/z. The main mass conditions were: capillary temperature 320 °C, source voltage 3.2 kV, sheath gas (35 arbitrary units), auxiliary gas (15 arbitrary unit) and sweep gas (10 arbitrary unit). Nitrogen (>99.98%) was employed as sheath gas, auxiliary and sweep gas. Instrument control and data acquisition were performed with Chameleon 6.8 software and Xcalibur 2.2 software (Thermo Fisher Scientific, San Jose, CA, USA).

### Statistical analysis

The results were express as mean ± S.E.M and analyzed using One-way Analysis of Variance (ANOVA), followed by Dunnett’s multiple comparison tests. Results were considered significant when *p* < 0.05.

## Results

### Antioxidant activity of MMMC at various ratios

**Fig. 1:**
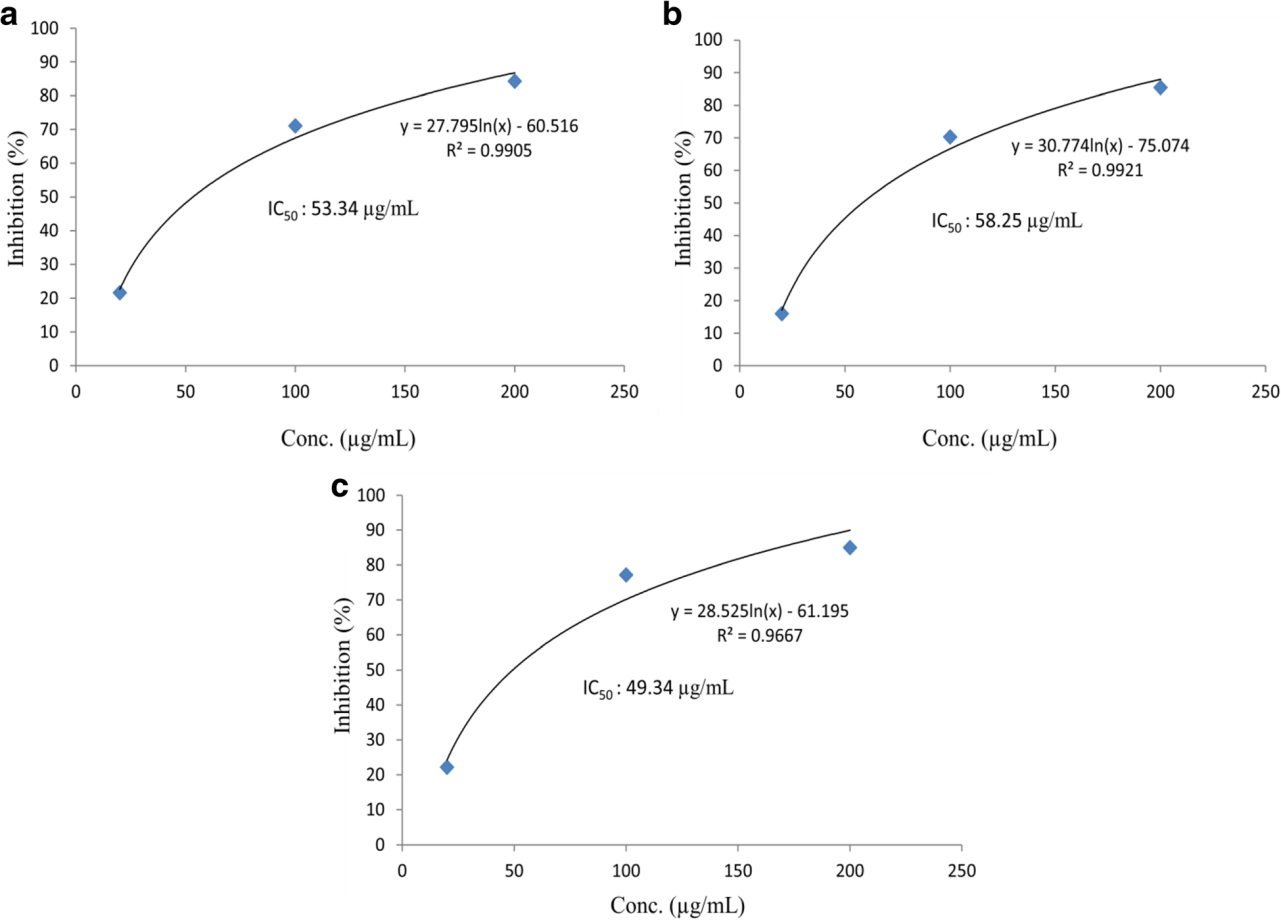
The IC_50_ value of MMMC at different ratio assessed using the DPPH scavenging assay. **a** 1:1 (*v*/v) MMMC. **b** 1:3 (v/v) MMMC. **c** 3:1 (v/v) MMMC

The antioxidant activity of MMMC at the ratio of 1:1, 1:3 and 3:1 were determined via DPPH radical scavenging, TPC and ORAC assays. The IC_50_ value for all MMMC against DPPH assay was 53.34, 58.25 and 49.34 μg/mL for ratio 1:1, 1:3 and 3:1 respectively (Fig. 1). Meanwhile, at the concentration of 200 μg/mL, MMMC at the ratio of 1:1 showed the highest value for TPC and ORAC assay in comparison to the other two ratios (Table 1).

**Table 1:** Total phenolic content and Oxygen radical absorbance capacity of MMMC

Sample	Extract (Ratio)	Concentration (μg/ml)	Total phenolic content (TPC) mg/100 g GAE	ORAC (μM TE/100 mg)
Control			Gallic acid (GAE) standard curve	Trolox standard curve
MMMC	1:1	200	379.0 ± 5.13	30,256 ± 2700
	1:3	200	279.3 ± 3.28	22,881 ± 3100
	3:1	200	279.3 ± 1.20	11,957 ± 1100

All values are expressed as mean ± SEM

TPC 1. TPC value of > 1000 mg GAE/100 g is considered as having high total phenolic content

TPC 2. TPC expressed as miligram equivalent to gallic acid per 100 g of dry weight (mg GAE/100 g)

ORAC: ORAC value expressed as μM Trolox Equivalent (TE)/100 g, are mean values from triplicate wells in duplicate Experiments, with SEM < 20%

### Effect of MMMC at various ratios on the ethanol-induced gastric ulcer

**Fig. 2:**
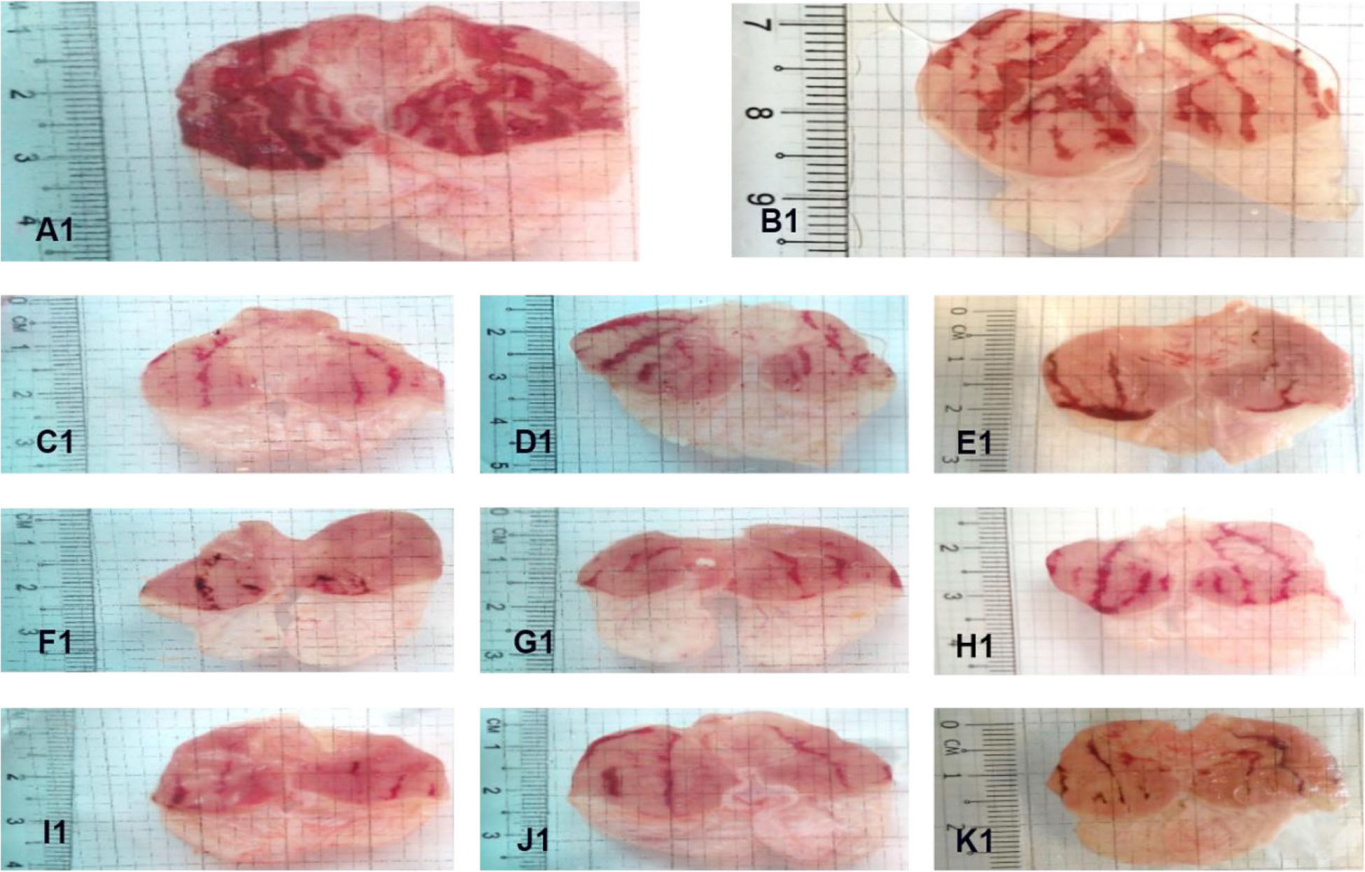
Gross examination of the ethanol intoxicated gastric mucosa tissue of rats orally pretreated with various test solutions. (A1) Rats pretreated with 2% Tween 80 (ulcer control). Severe lesions observed with extensive visible hemorrhagic necrosis of gastric mucosa. (B1) Rats pretreated with ranitidine (100 mg/kg, positive control). Moderate lesions of gastric mucosa were seen compared to the lesions in ulcer control group. (C1, D1, E1) Rats pretreated with low dose of 1:1, 1:3 and 3:1 MMMC, respectively (15 mg/kg). Very moderate lesions were seen in group the D1 and E1. Meanwhile, group C1 shows mild lesions. (F1, G1, H1) Rats pretreated with 150 mg/kg of 1:1, 1:3 and 3:1 MMMC respectively. Group F1 and G1 exhibit mild lesions when compared to the group treated with 2% Tween 80. Very moderate lesion was formed in group H1. (I1, J1, K1) Rats pretreated with high dose of 1:1, 1:3 and 3:1 MMMC (300 mg/kg). Very moderate lesion was seen in group K1. Meanwhile, group I1 and J1 shows a very mild lesion, which indicates highly protection of the extract against gastric ulcers

**Fig. 3:**
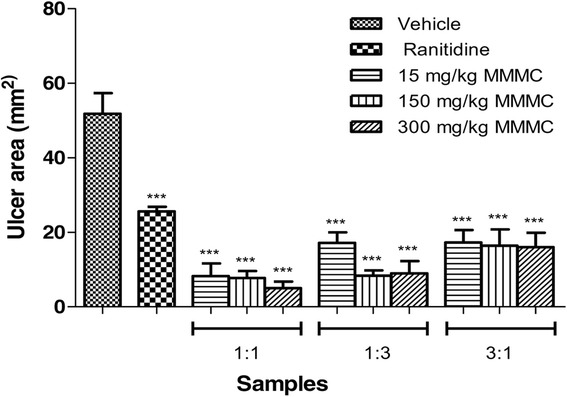
Effect of orally administered vehicle (Tween 80, 2%), ranitidine (100 mg/kg) or different ratio of 15, 150 and 300 mg/kg MMMC on ulcer area formation in the ethanol-induced gastric ulcer model. The ulcerated area (mm^2^) was expressed as Mean ± SEM for six animals. One way ANOVA was followed by Dunnett’s post-hoc test, ***p < 0.001 vs vehicle

Administration of the absolute ethanol solution to the control group obviously produced the characteristics necrotic lesions expected (Fig. 2) Gastric lesion measurements of ethanol-intoxicated rats showed that MMMC in the ratio of 1:1 and 3:1 significantly (*p* < 0.001) prevent the ulcer formation at all doses tested in a dose-dependent manner (Fig. 3). The percentage of protection range recorded by 1:1 MMMC was approximately 84-91% while for the 3:1 MMMC, it was 67-70%. On the other hand, 1:3 MMMC exerted a significant (*p* < 0.001) gastroprotection in a dose-independent manner with the percentage of protection recorded in the range of 67-83%. At the lowest dose (15 mg/kg), the highest protection percentage was showed by ratio 1:1, followed by 1:3 and 3:1. Ranitidine (100 mg/kg; positive control) also showed significant (*p* < 0.001) prevention of gastric ulcer formation with the recorded percentage of protection of approximately 51%.when compared to the control group.

### Histopathological findings of the ethanol-induced gastric ulcer tissues pretreated with MMMC at various ratios

**Fig. 4:**
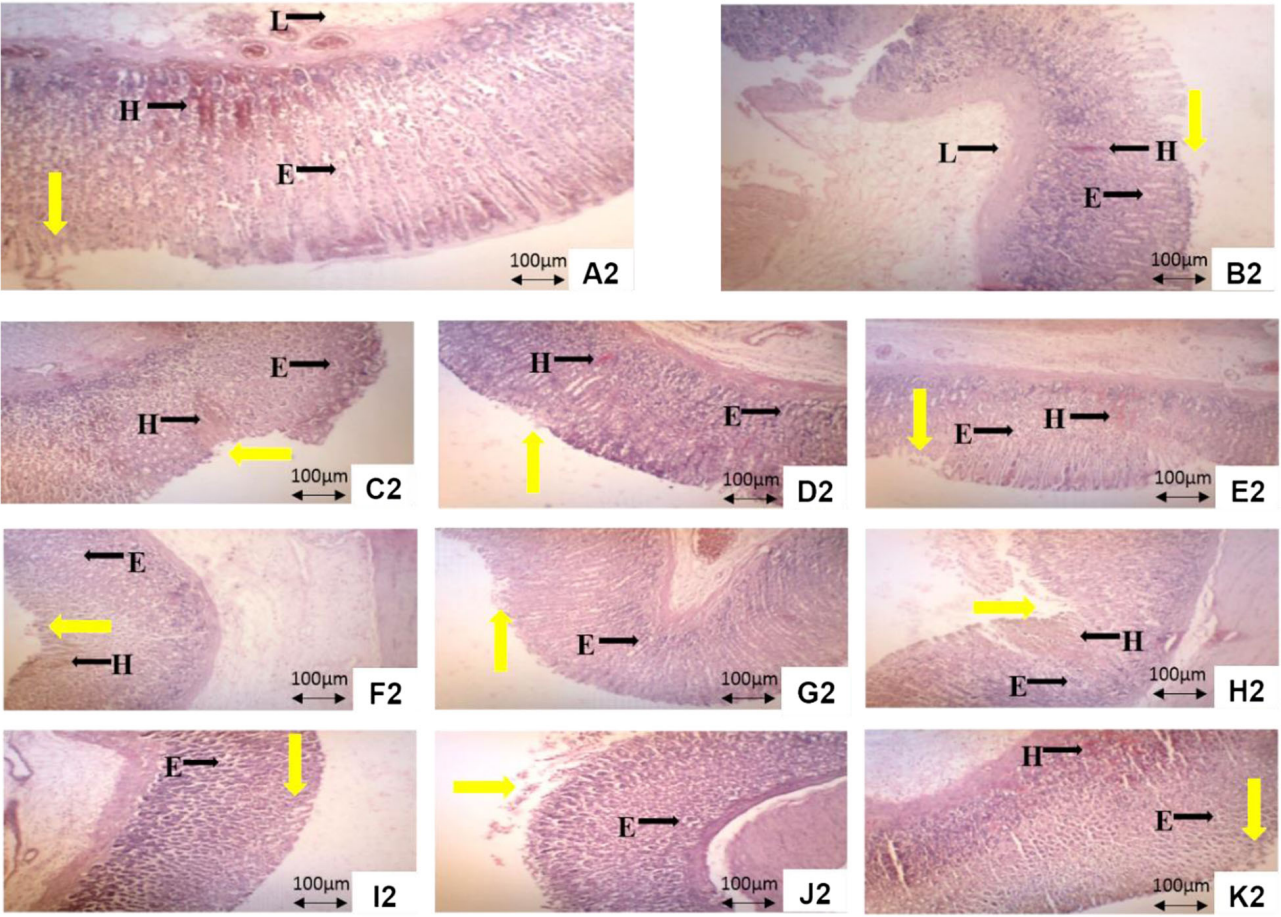
Histological evaluation of the ethanol intoxicated gastric mucosa tissue of rats orally pretreated with various test solutions. (A2) Rats pretreated with 2% Tween 80 (ulcer control) showing severe destruction to surface epithelium and necrotic lesions. (B2) Rats pretreated with ranitidine (100 mg/kg, positive control). Moderate disruption to gastric mucosa layer with moderate edema and hemorrhage, in addition of leukocyte infiltration was observed. (C2, D2, E2) Stomach receiving low dose of 1:1, 1:3 and 3:1 MMMC, respectively (15 mg/kg). Mild lesion on mucosa was seen in group C2 with mild hemorrhage and edema. Meanwhile, in group D2 and E2 show a very moderate effect on mucosa with mild hemorrhage and edema. (F2, G2, H2) Stomach pretreated with 150 mg/kg of 1:1, 1:3 and 3:1 MMMC respectively. Mild effect on mucosa and edema were formed in group F2 and G2. Group H2 exhibit very moderate lesion and mild edema and hemorrhage. (I2, J2, K2) Stomach pretreated with 300 mg/kg of 1:1, 1:3 and 3:1 MMMC with group K2 showing a moderate effect on mucosa with moderate hemorrhage and mild edema. Group I2 exhibit very mild effect mucosa with edema indicating that the 1:1 MMMC at its highest dose was highly able to protect the gastric mucosa. The yellow arrow indicates disruption to the surface epithelium. H-hemorrhage; E-edema; L-leukocyte infiltration (H&E staining, 10 x)

Histopathological observation of ethanol-induced gastric ulcer tissue in control group showed severe lesions and extensive damage to the gastric mucosa. Severe hemorrhage, edema, leukocytes infiltration, necrosis, and ruination of the surface epithelium and necrotic lesions penetrating deeply into mucosa were observed (Fig. 4). Groups pre-treated with 1:1 MMMC, at all doses, and 1:3 MMMC, at 150 and 300 mg/kg, showed relatively better protection of gastric mucosa indicated by reduction in ulcer area, reduced or nonappearance of hemorrhage and necrosis, with mild or none submucosal edema and minor or no disruption to the surface epithelium and deep mucosa (Fig. 4). On the other hand, pre-treatment with 1:3 MMMC, at its lowest dose, and 3:1 MMMC, at all doses, demonstrated mild lesion of the mucosa with moderate to mild effects of hemorrhage and edema similar to the ranitidine pre-treated group (Fig.4).

### Effect of MMMC at various ratios on various parameters of gastric ulcer assessed using the pylorus ligation assay

#### Effect of MMMC at various ratios on volume, pH and, free and total acidity of gastric juice content

The effects of MMMC upon baseline acid secretion collected after 6 h of pylorus ligature in rats are shown in Table 2. The 1:1 and 1:3 MMMC caused significant (*p* < 0.001) decreased in the volume of gastric secretion at all doses tested with the percentage of inhibition recorded ranging between 43 and 56% and 44-52%, respectively. However, the later occured in a dose-independent manner. For the 3:1 MMMC, only doses at 150 and 300 mg/kg caused significant (*p* < 0.001) decreased in the volume of gastric secretion. As for the pH of the gastric juice, only 1:1 MMMC caused significant (*p* < 0.05) increased in this parameter at all doses tested with the 1:3 MMMC caused insignifcant (*p* > 0.05) changed to the pH level of the gastric juice. On the other hand, the 3:1 MMMC only affected the gastric juice’s pH significantly (*p* < 0.001) at the dose of 150 mg/kg. With regard to the total acidity of the gastric juice, only 300 mg/kg 1:1 MMMC, 150 and 300 mg/kg 1:3 MMMC and 150 mg/kg 3:1 MMMC caused significant (*p* < 0.001) reduction in the said parameters. Ranitidine, at 100 mg/kg, significantly (*p* < 0.05) reduced the volume of gastric juice by approximately 30% and significant (*p* < 0.001) decreased the total acidity of the gastric juice while significantly (*p* < 0.05) increased the pH of gastric juice by almost 2.6 fold when compared to the control group (2% Tween 80).

**Table 2:** Effect of MMMC on several gastric content parameters assessed using the pylorus-ligated rat model

Pre-treatment	Ratio	Dose (mg/kg)	Volume of gastric juice (ml)	pH	Total acidity (mEq/L)
2% Tween 80		–	4.58 ± 0.375	1.57 ± 0.428	664.0 ± 31.79
Ranitidine		100	3.25 ± 0.250*	4.13 ± 0.632***	386.7 ± 24.59***
MMMC	1:1	15	2.58 ± 0.301***	1.98 ± 0.319	600.0 ± 61.97
		150	2.50 ± 0.183***	2.32 ± 0.111*	710.0 ± 35.68
		300	2.08 ± 0.352***	2.38 ± 0.120*	466.7 ± 24.59**
	1:3	15	2.17 ± 0.211***	1.38 ± 0.294	540.0 ± 58.20
		150	2.17 ± 0.279***	1.90 ± 0.608	370.0 ± 15.28***
		300	2.58 ± 0.375***	2.03 ± 0.328	266.7 ± 19.78***
	3:1	15	4.08 ± 0.271	1.77 ± 0.163	633.3 ± 31.69
		150	2.75 ± 0.214***	3.85 ± 0.264***	306.7 ± 30.40***
		300	1.83 ± 0.247***	1.92 ± 0.409	590.0 ± 45.46

Values are expressed as mean ± SEM for six animals in each group. One way ANOVA was followed by Dunnett’s post hoc test

*p < 0.05 as compared to the control group within the respective column

**p < 0.01 as compared to the control group within the respective column

***p < 0.001 as compared to the control group within the respective column

### Effect of MMMC at various ratios on the releases of gastric wall mucus content

**Fig. 5:**
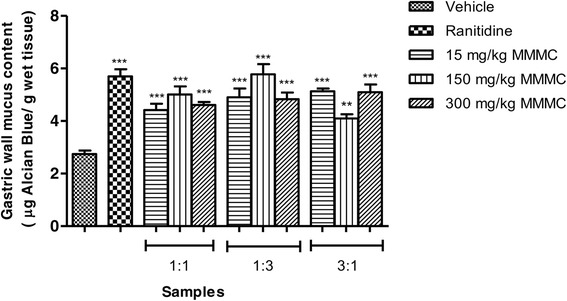
Effect of orally administered vehicle (Tween 80, 2%), ranitidine (100 mg/kg) or, different ratio of 15, 150 and 300 mg/kg MMMC on gastric wall mucus production in the ethanol-induced gastric ulcer model. The gastric wall mucus content (μg Alcian Blue/g wet tissue) was expressed as Mean ± SEM for six animals. One way ANOVA was followed by Dunnett’s post-hoc test, ***p < 0.001, **p < 0.01 vs vehicle

As observed in Fig. 5, pre-treatment with MMMC, at all ratios and doses, caused significant (*p* < 0.001) increase in the gastric wall mucus content. The amount of mucus recorded for each ratio of MMMC was significantly higher (*p* < 0.001) when compared against the control group that received vehicle alone. The rats that received 100 mg/kg ranitidine also increased the mucus content significantly (*p* < 0.001).

### Effect of MMMC at various ratios on CAT, SOD and GSH activities and MDA level in the ethanol-induced gastric ulcer tissue

The effect of MMMC on CAT activity, SOD, GSH and MDA level of activity upon ethanol-induced stomach tissue homogenates were presented in Table 3. The result showed a significant decreasing in the level of SOD and GSH as well as CAT activities after the ethanol oral administration. The MMMC significantly ascended (*P* < 0.001) the activity of CAT and level of GSH at all of the three ratios (50, 150 and 300 mg/kg) together with ranitidine group. Meanwhile, SOD level showed to be increased at all of the three ratios and doses of MMMC but only significant for two doses (150 and 300 mg/kg) of ratio 1:1, 1:3 and 3:1 of MMMC. Meanwhile, upon ethanol administration, the level of MDA seems to be increased when compared to the normal group. However, this value was decreased by the 1:1 ratio of MMMC treatment group, while the other two groups of 1:3 and 3:1 ratio of MMMC failed to reduce the level of MDA. Therefore, the result obtained showed that ratio 1:1 of MMMC was the most successful group of treatment that can restore the changes of antioxidant mechanisms upon ethanol induction.

**Table 3:** Antioxidant activity of MMMC against ethanol-induced gastric ulcer in stomach tissue of rats

Pre-treatment	Ratio	Dose (mg/kg)	CAT (nmol/min/mL)	SOD (U/mg protein)	GSH (μM/mg protein)	MDA (ng/mL)
Normal		–	60.47 ± 2.251	4.72 ± 0.364	43.28 ± 1.118	11.42 ± 0.577
2% Tween 80		–	44.11 ± 1.591***	1.56 ± 0.240***	6.606 ± 1.175***	75.00 ± 1.732***
Ranitidine		100	66.11 ± 1.569***	3.20 ± 0.381*	23.68 ± 1.037***	15.00 ± 1.693***
MMMC	1:1	15	66.27 ± 1.439***	2.60 ± 0.469*	35.61 ± 1.651***	33.07 ± 2.214***
		150	67.01 ± 1.579***	3.94 ± 0.351***	35.80 ± 0.578***	31.44 ± 1.605***
		300	69.54 ± 1.846***	3.96 ± 0.365***	38.48 ± 0.955***	26.21 ± 1.707***
	1:3	15	68.09 ± 1.932***	2.70 ± 0.327*	39.46 ± 1.878***	46.11 ± 0.824**
		150	66.58 ± 2.120***	3.65 ± 0.222***	38.47 ± 0.716***	36.11 ± 1.883***
		300	63.51 ± 2.016**	3.46 ± 0.361**	30.49 ± 1.280***	35.83 ± 2.455***
	3:1	15	69.03 ± 1.449***	2.66 ± 0.372*	32.27 ± 1.196***	63.06 ± 2.544*
		150	68.33 ± 1.456***	3.79 ± 0.329***	42.22 ± 0.705***	41.11 ± 1.321***
		300	64.51 ± 1.965**	3.82 ± 0.362***	33.36 ± 1.224***	38.22 ± 1.833***

Data are presented as mean ± S.E.M. Sixty rats (*n* = 6 in each group) were used in this study. Statistical analysis was performed

using the one-way ANOVA followed by Dunnet’s multiple comparison tests. *P < 0.05,**P < 0.01 and ***P < 0.001 as compared to ulcer control group

### Effect of MMMC on PGE_2_ level in the stomach tissue of ethanol treated rats

When compared to the normal group, the result obtained from the ulcer control group had proved that reduction of PGE_2_ production was due to ethanol induction. The treatment of the highest dose (300 mg/kg) of all the three ratios of MMMC was significantly (*P* < 0.001) ascended the value of PGE_2_ (Table 4). All of the ratio treatment groups showed a dose-dependent manner of increasing of the PGE_2_ level, even though the lowest dose (15 mg/kg) of those groups did not markedly prove the improvement.

**Table 4:** Effect of MMMC on the PGE2 content in ethanol-induced gastric tissue of rats

Pre-treatment	Ratio	Dose (mg/kg)	PGE_2_ (pg/mL)
Normal		–	188.10 ± 1.70
2% Tween 80		–	159.20 ± 0.93*
Ranitidine		100	186.40 ± 4.93***
MMMC	1:1	15	174.30 ± 2.65
		150	188.40 ± 4.40***
		300	193.40 ± 5.98***
	1:3	15	173.40 ± 3.93
		150	183.40 ± 3.43***
		300	192.40 ± 4.72***
	3:1	15	162.30 ± 5.79
		150	172.20 ± 2.73
		300	185.20 ± 3.34***

Data are presented as mean ± S.E.M. Sixty rats (*n* = 6 in each group) were used in this study. Statistical analysis was performed using the one-way ANOVA followed by Dunnet’s multiple comparison tests. *P < 0.05,**P < 0.01 and ***P < 0.001 as compared to ulcer control group

### Profiling of the phenolic compounds in 1:1 (*v*/v) MMMC

**Fig. 6:**
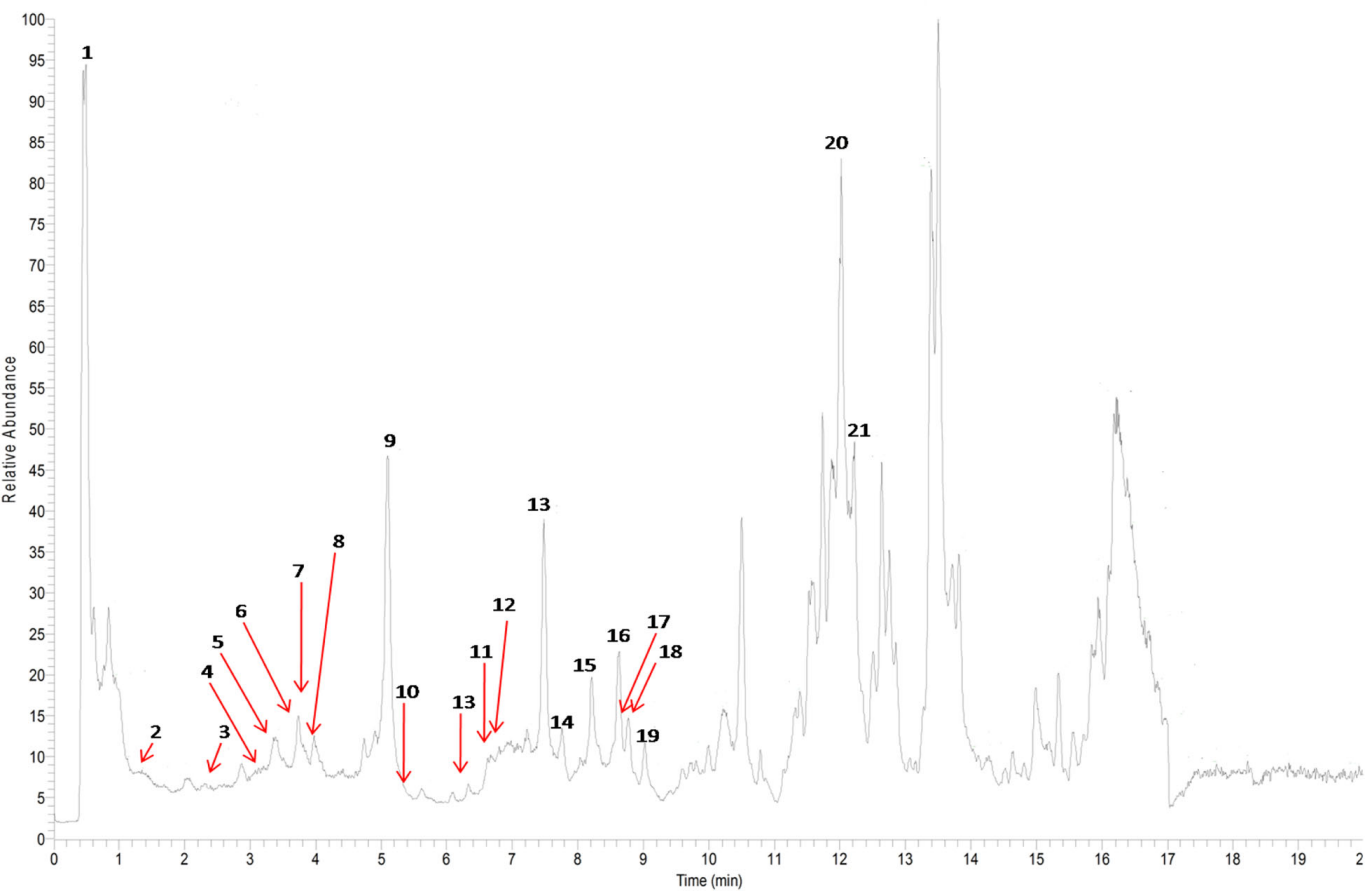
UHPLC analysis of MMMC in negative ion mode. Total ion chromatography (TIC) profile of the 1:1 (v/v) MMMC

The profiling of phenolic compounds was performed using an UPLC–ESI–MS/MS instrument as described above only on the most effective MMMC, which was the 1:1 (v/v) MMMC. The extract was analysed based on the accurate mass data of the molecular ions using the data analysis software (Xcalibur 2.07, Thermo Fisher, San Jose, CA, USA).which issued the list of possible elemental formulas. Thus, based on the obtained base peak chromatogram, various peaks were detected of which 21 peaks were identified (Fig. 6). Compounds were identified through co-injection with reference samples available in the laboratory or on the basis of fragmentation patterns compared with literature data. The widely accepted precision threshold for confirmation of elemental compositions was established at 5 ppm. Table 5, on the other hand, list the peak number, retention time, observed *m/z*, the produced molecular formula and the detected compound.

**Table 5:** Flavonoids and phenolic compounds identified in MMMC (ratio 1:1) using the UHPLC-ESI-MS/MS

Peak No	t^R^ (min)	[M-H]-	Error (ppm)	Formula	Identification
1.	0.61	169.01389	4.971	C_7_H_5_O_5_	Gallic acid
2.	1.41	337.09412	6.90	C_16_H_17_O_8_	3-p-Coumaroylquinic acid
3.	2.30	163.04001	6.375	C_9_H_7_O_3_	Coumaric acid
4.	3.41	300.999	5.071	C_14_H_5_O_8_	Ellagic acid
5.	3.52	609.14819	5.120	C_27_H_29_O_16_	Quercetin-3-rutinoside
6.	3.73	463.08984	5.912	C_21_H_19_O_12_	Quercetin-3-glucoside 2
7.	3.81	431.09946	5.073	C_21_H_19_O_10_	Vitexin
8.	3.97	463.08978	5.782	C_21_H_19_O_12_	Quercetin-3-glucoside 1
9.	5.10	447.09607	4.859	C_21_H_19_O_11_	Quercetin rhamnoside Orientin
10.	5.35	317.03094	5.508	C_15_H_9_O_8_	Myricetin
11.	6.62	461.11047	5.708	C_22_H_21_O_11_	Scoparin (Chrysoeriol-8-C-glucoside)
12.	6.62	461.11050	5.773	C_22_H_21_O_11_	Isoharmnetin-3-O-rhamnoside
13.	6.32	431.09933	4.771	C_21_H_19_O_10_	Isovitexin
14.	7.34	609.12708	5.251	C_30_H_25_O_14_	Prodelphinidin B3
15.	7.49	301.03595	5.551	C_15_H_9_O_7_	Quercetin
16.	8.20	593.13196	5.046	C_30_H_25_O_13_	Kaempferol-3-(p-coumarylglucoside) 1
17.	8.53	593.13184	4.844	C_30_H_25_O_13_	Kaempferol-3-(p-coumarylglucoside) 2
18.	8.63	271.06116	3.911	C_15_H_11_O_5_	Narigenin
19.	9.02	285.04062	4.405	C_15_H_9_O_6_	Luteolin
20.	12.02	299.05606	3.496	C_16_H_11_O_6_	Diosmetin
21.	12.22	269.04550	2.677	C_15_H_9_O_5_	Apigenin

## Discussion

Previous studies have reported on the antiulcer individually activity of methanolic leaves extract of *M. malabathricum* [22] and *M. calabura* [23] against the ethanol-induced ulcer assay when tested individually. In these studies, the dose range used was 50-500 mg/kg. The MEMM did not exert ameliorative effect against ethanol/ intoxication at doses of 50 and 250 mg/kg whereas MEMC, at the same doses, demonstrated significant antiulcer activity with the recorded percentage of ulcer inhibition of approximately 60% and 80%, respectively. At the dose of 500 mg/kg, both extracts caused more than 90% ulcer inhibition.

Since both plants possess antiulcer potential and in an attempt to develop a pharmaceutical product from them, we took the opportunity to study the synergistic effect of a combination of these plants (MMMC) at different ratio on their antiulcer potential using various animal models. In the present study, the dose range used was between 15 and 300 mg/kg, which is lower than the dose range used for both plant when studied separately. MMMC administration showed a significant protection against ethanol-induced gastric ulcer at different ratio used. Interestingly, MMMC, at all ratio tested, caused remarkable antiulcer activity at 15 mg/kg (the lowest dose used) with the most effective extract, 1:1 (*v*/v) MMMC, exhibited approximately 80% ulcer inhibition. At 150 and 300 mg/kg, 1:1 (v/v/) MMMC exerted significant gastroprotective activity that was similar to 500 mg/kg MEMM or 100-500 mg/kg MEMC. Taking into consideration the previous and present findings as described above, it is plausible to suggest the presence of synergistic action between the two plants, which help to improve the gastroprotective action of MMMC in comparison to MEMM or MEMC.

Moreover, all ratios of MMMC promote a more effective gastroprotective effect when compared to ranitidine, the positive antiulcer drug, possibly by triggering the local and non-specific mechanism known as adaptive cytoprotection, which ameliorates the aggressive factors effect while increases the defensive factors, thus, shields the gastric mucosa from damage [22]. Nevertheless, the dose-independent antiulcer effect of 1:3 (*v*/v) MMMC can be related to the ‘therapeutic windows’ effect as stated by Tripathi [32]. Reduction in a drug’s potential can be due to the presence of high concentration of its active principle [32]. Hence, to reach its maximum curative effect, a particular drug has to be within its therapeutic window. With regard to 1:3 (*v*/v) MMMC, it is suggested that the dose 150 mg/kg was within the ‘therapeutic windows’ of that mixture, which might explain the highest antiulcer activity observed using the ethanol-induced gastric ulcer model. On the other hand, the dose 300 mg/kg, which showed reduction in the antiulcer effect, might indicate that this dose has already been outside the ‘therapeutic windows’ of the respective MMMC. In addition, the ability of MMMC to attenuate the ethanol-induced gastric ulcer was further supported by the microscopic findings.

The extract was also able to modulate several parameters of the gastric juice secretion and the gastric wall mucus secretion level when assessed using the pyloric ligation model. In the present study, i) all doses of 1:1 and 1:3 (v/v) MMMCs reduced the volume of gastric secretion, ii) only 1:1 (v/v) MMMC, at all doses, increased the pH of gastric secretion, and, iii) only 300 mg/kg 1:1 (v/v) MMMC, 150 and 300 mg/kg 1:3 (v/v) MMMC and 150 mg/kg 3:1 MMMC reduced the total acidity of gastric secretion. These findings suggested that MMMC worked to protect the gastric mucosal from injury by reducing the volume and total acidity while increasing the pH of the gastric juice secretion. In addition, all ratios of MMMC, at all doses tested, increased the gastric wall mucus content. Gastric mucus plays a crucial role in the gastric ulcer defense mechanism, whereby it forms a continuous mucus gel-like protective barrier that covers the entire gastric mucosa and keeps the mucosal surface at a pH of 6-7 in the acidic environment. Venables [33] claimed that the increase in amount of mucus secreted by the gastric mucosal cells can help to avoid ulcer formation by playing a role as an effective barrier to the back diffusion of hydrogen ions, enhancing the buffering of gastric acid juice and descending stomach wall friction throughout peristalsis. The present study validates the fact that one of the potential mechanisms of gastroprotective effect demonstrated by MMMC involves strengthening of the gastric mucosal protection via increase gastric mucus secretion.

Other than the ability of MMMC to modulate the imbalance between aggressive and defensive factors that strongly contributes to the ulcer formation, the extract ability to adjust the level of oxidative process and to reduce the presence of free radicals may also play a vital role in protecting the gastric mucosa from ulcer formation [34]. Ethanol has been reported to promote the production of free radicals like reactive oxygen species (ROS) and at the same time impedes the body’s normal defense mechanisms against the action of those ROS through numerous processes, especially in the liver [35]. For instances, i) ethanol breakdown in the liver leads to the formation of molecules whose further metabolism in the cells leads to ROS production; ii) ethanol also excites the activity of cytochrome P450s, enzymes that are found in the liver, resulting in the ROS production; iii) ethanol facilitates ROS production by altering the levels of certain metals in the body; or, iv) ethanol decreases the levels of mediators such as antioxidants, which involve in the elimination of ROS. ROS can injure or cause complete deprivation (i.e., peroxidation) of fundamental complex molecules in the cells, including fat molecules (i.e., lipids), proteins, and DNA. Due to the excessive formation and presence of ROS following the acute and chronic exposure to ethanol, enhancement of lipids, protein and DNA peroxidation take place in the cells resulting in the cells being transfer to the state known as oxidative stress, which if untreated can result in cells injury [36]. It is suggested that the tendency of an extract/compound to scavenge free radicals and exhibit antioxidants characteristics could also be one of the important mechanisms via which the extract/compound exerts its gastroprotective effect. According to Trivedi and Rawal [34], oxidative stress takes vital part in the pathogenesis of gastric ulcer and suggests that antioxidant agents play important role by providing protection to the gastric mucosa against various necrotic agents. In line with these claims, the present study revealed the high antioxidant capacity of MMMC, at all ratios tested, when assessed using the DPPH radical scavenging and ORAC assays. DPPH radical scavenging assay is a rapid, simple, inexpensive and widely used method to measure the ability of compounds to act as free radical scavengers or hydrogen donors, and to evaluate antioxidant activity of foods [37]. The ability of all ratios of MMMC to remarkably scavenged free radicals when assessed using the DPPH radical scavenging assay could be based on the IC_50_ value recorded that range between 49 and 58 μg/mL. Various reports on antioxidant activity of different extracts of a diverse range of plants have been published elsewhere. Taking into account that some plants were considered to show stronger radical-scavenging abilities with the recorded IC_50_ ranging between 4 and 185 μg/mL whereas others were assumed to demonstrate much weaker radical-scavenging abilities with the recorded IC_50_ ranging between 350 and 2905 μg/mL [35], the present study revealed that MMMC, at all ratios, possess strong radical scavenging action. Furthermore, MMMC also demonstrated significant activity when assessed using the ORAC assay at all ratio tested in the sequence of 1:1, 1:3 and 3:1 MMMC. The ORAC assay measures the peroxyl radical scavenging capacity by measuring the ability of potential antioxidants to inhibit the fluorescein oxidation by peroxyl radicals. According to Turner et al. [38], high reading of ORAC indicated a significant or strong peroxyl radical absorbing capacity of the extract. Peroxyl radical is a reactive species mainly involved in the propagation step of lipid peroxidation. It is plausible to suggest from this observation that MMMC may also be able to help prevent the process of lipid peroxidation during ulcer formation. It is interesting to note that there is a synergistic action between the different bioactive compounds in both plants that help to preserve the antioxidant potential of MMMC as assessed using both the DPPH radical scavenging and ORAC assays.However, it is also observed that the antioxidant activity of MMMC depends on the ratio used as increase in the amount of *M. calabura* in comparison to *M. malabathricum* as seen with the 1:3 MMMC shows low antioxidant activity in both antioxidant assays. Moreover, the high antioxidant activity of MMMC might also be associated with its high TPC value indicating the presence of high polyphenolic compounds. Polyphenolic compounds have been reported to demonstrate remarkable antioxidant and anti-inflammatory activities, which might contribute to the observed antiulcer activity of MMMC [39].

It is worth to highlight again on the ability of ethanol, in this case, to obstruct the normal defense mechanisms of the body against ROS activity. The human body has several mechanisms to thwart oxidative stress by producing endogenous or exogenous antioxidants [40]. The former are naturally produced in situ while the latter are supplied through foods and/or supplements. The roles of these antioxidants are to neutralize the excess of free radicals, to protect the cells against their toxic effects and to contribute to disease prevention by acting as free radical scavengers. Endogenous antioxidants in cells can be clategorized as enzymatic and non-enzymatic antioxidants. Some of the major endogenous antioxidant enzymes widely studied for their ability to neutralize ROS and RNS include superoxide dismutase (SOD), catalase (CAT) and glutathione peroxidase (GSH). On the other hand, the non-enzymatic antioxidants are also divided into metabolic antioxidants and nutrient antioxidants. The metabolic antioxidants are produced through metabolism in the body such as glutathione, coenzyme Q10, bilirubin and transferrin. On the other hand, the nutrient antioxidants are compounds which cannot be produced in the body and must be provided through foods or supplements such as flavonoids, vitamin C and omega-3 and omega-6 fatty acids [41]. In the present study, the defense role played by endogenous antioxidant enzymes particularly SOD and CAT on the gastric ulcer tissue was studied. The MMMC, at all ratios and doses tested, reversed the ethanol-induced reduction in the level of SOD and CAT indicating that the MMMC exerts its gastroprotective effect partly via modulation of the gastric endogenous antioxidant system. The ability of MMMC to modulate endogenous enzymatic antioxidants system could be attributed to the respective MEMM or MEMC potentials to activate the same endogenous defense system separately [22, 23]. The involvement of endogenous non-enzymatic antioxidant in the modulation of ulcer formation by MMMC was also determined by measuring the level of GSH in the ethanol-induced gastric ulcer tissue. GSH is found abundantly in gastric mucosa and helps to maintain the integrity of mucosal as well as discharge H_2_O_2_ and superoxide anions that may worsen the tissue damage [42]. In the present study, MMMC, at all ratios and doses, caused remarkable increase in the level of GSH in the gastric tissue, which was reduced by ethanol administration.

The effectiveness of MMMC was further supported by the high TPC value, which indicates the presence of high polyphenolic compounds such as flavonoids and tannins. The presence of high flavonoids and tannins, in particular, in MMMC could be explained by the previous findings on the phytochemical contents of MEMM or MEMC, respectively [22, 23]. Oxidative stress, generated by the excess ROS and oxidants, is a deleterious process that can seriously alter the cell membranes and other structures such as proteins, lipids, lipoproteins, and DNA [43]. For example, excess formation of hydroxyl radical and peroxynitrite can damage cell membranes and lipoproteins by a process called lipid peroxidation. This reaction results in the formation of MDA and conjugated diene compounds, which are cytotoxic and mutagenic. Lipid peroxidation spreads rapidly and affects a large number of lipid molecules. MDA is the final product of lipid peroxidation, which is widely used as an indicator for determination of lipid peroxidation level. In addition, free radicals attack on proteins leads to structural changes and loss of enzyme activity [41]. One of the mechanisms used by the body to counteract these attacks is by using the antioxidants defense system. In the present study, MMMC, at all ratios and doses used, remarkably reversed the ethanol-induced increase in MDA level. Thus, suggesting the potential of MMMC to attenuate lipid peroxidation in the gastric tissue.

Other than that, the role of prostaglandins (PGs), particularly PGE_2_, in protecting the gastric mucosa layer from gastric ulcer in the presence of MMMC was also investigated. PGs, particularly PGE_2_, also have strong cytoprotective effects on the gastric mucosa as a result of various indirect mechanisms that include inhibition of acid secretion, amelioration of mucosal blood flow, increased epithelial mucus production and bicarbonate secretion, inhibition of gastric motility, inhibition of free radical and enzyme release from neutrophils, vascular, luminal, and/or extrinsic and intrinsic neural mechanisms, and direct protection of gastric mucosal cells from various gastric irritants [44, 45]. In the present study, ethanol was found to reduce the concentration of PGE_2_ and pretreatment with MEMC, at all ratios and doses, prevents the ethanol-induced decrease in PGE_2_concentration. These findings further suggest the important of PGE_2_ presence in the maintenance of the gastric mucosa layer and the ability of MMMC to preserve the existence of PGE2 in the presence of gastric irritants.

The gastroprotective potential of MMMC could also be attributed to the presence of polyphenols particularly flavonoids [22, 23]. Several articles have reported that flavonoids possess antiulcer activity [46, 47]. The UHPLC-ESI-MS analysis of MMMC revealed the presence of 21 flavonoid-based bioactive compounds. Of these, several flavonoids have been reported to exert antiulcer activity such as gallic acid [48], coumaric acid [49], ellagic acid [50] and quercetin [51] to name a few, and is, therefore, believed to act synergistically to demonstrate the antiulcer activity. The mechanisms of antiulcer activity demonstrated by some of these flavonoids were discussed below. According to Abdelwahab et al. [48] and Sen et al. [52], the main mechanisms of antiulcer action of gallic acid involved its effect on: i) gastric acid secretion, ii) promotion of mucosal protection by activating the endogenous factors (NO, PGE2 and tumor necrosis factor-α), iii) prevention of proinflammatory cytokines production, iv) inhibition of oxidative stress-induced apoptosis, v) inhibition of histamine release from mast cells, vi) increasing the mucosal defensive factors, and vii) activating the antioxidant mechanisms. On the other hand, ellagic acid exerts antiulcer activity via the: i) inhibition of gastric H+, K + −ATPase, ii) inhibition of acid secretion, and iii) attenuation of lipid peroxidation in the gastric mucosa and myeloperoxidase in the intestinal mucosa of ethanol-induced ulcer model [50, 53]. Moreover, quercetin have been reported to exert antiulcer activity via mechanisms involving: i) inhibition of lipid peroxidation, ii) reduction of protein carbonyl compounds, iii) increase in the levels of mucosal non-protein SH compounds, iv) increase in glutathione peroxidase activity, v) enhancement of the SOD activity, vi) reduction of the acid production and inhibition of the H^+^/K^+^-ATPase activity, and vii) amelioration of ROS production [54–56]. It is, therefore, plausible to suggest that the synergistic effect of these bioactive compounds might contribute to the gastroprotective activity of MMMC.

## Conclusion

In conclusion, the present study shows the ability of all ratios of MMMC to markedly ameliorate gastric ulceration in ethanol intoxicated rats with 1:1 (*v*/v) MMMC demonstrated the most effective gastroprotective activity. Moreover, the ability of MMMC to reduce the gastric secretion and total acidity while increasing the mucus production explains the balance protection of MMMC against the aggressive and defensive factors of gastric ulcer. The synergistic action between *M. malabathricum* and *M. calabura* is also proposed based on MMMC ability to trigger gastroprotection at the lowest dose that was ineffective when compared against MEMM or MEMC, in which each plant was tested indicidually.

## Abbreviations

ANOVA: One-way analysis of variance
cAMP: Cyclic adenosine monophosphate
CAT: Catalase
DPPH: Diphenyl-1-picrylhydrazyl
GAE: Gallic acid equivalent
GSH: Glutathione
H_2_O_2_: Hydrogen peroxide
HCl: Hydrochloric acid
IACUC: International animal care and used committee
*M. calabura*: *Muntingia calabura*
*M. malabathricum*: *Melastoma malabathricum*
MDA: Malondialdehyde
MMMC: *Melastoma malabathricum Muntingia calabura*
NaOH: Sodium hydroxide
NO: Nitric oxide
OD: Optical density
ORAC: Oxygen radical absorbance capacity
PGE_2_: Prostaglandins E_2_
PGs: Prostaglandins
RNS: Reactive nitrogen species
ROS: Reactive oxygen species
rpm: Revolutions per minute
S.E.M: Standard error of mean
SH: Sulfhydryl
SOD: Superoxide dismutase
TPC: Total phenolic content
UA: Ulcer area
UHPLC-ESI: Ultra high performance liquid chromatography-electrospray ionization
